# Closing gaps in histoplasmosis: clinical characteristics and factors associated with probable/histoplasmosis in HIV/AIDS hospitalized patients, a retrospective cross-sectional study in two tertiary centers in Pereira, Colombia

**DOI:** 10.1186/s12981-021-00377-5

**Published:** 2021-08-12

**Authors:** Julián Andrés Hoyos Pulgarin, John Alexander Alzate Piedrahita, German Alberto Moreno Gómez, Juan Felipe Sierra Palacio, Karen Melissa Ordoñez, Deving Arias Ramos

**Affiliations:** 1 Infectious Diseases, Pereira, Colombia; 2 Internal Medicine Physician, Pereira, Colombia; 3 Public Health Doctor, Pereira, Colombia; 4 General Physician, Pereira, Colombia; 5 Universidad Tecnológica de Pereira, Pereira, Colombia; 6 San Jorge University Hospital, Pereira, Colombia; 7 Grupo de Investigación en Medicina Interna, Universidad Tecnológica de Pereira, Pereira, Colombia

**Keywords:** HIV, AIDS, Histoplasmosis, Opportunistic mycosis, Risk factors

## Abstract

**Background:**

The HIV pandemic continues to cause a high burden of morbidity and mortality due to delayed diagnosis. Histoplasmosis is prevalent in Latin America and Colombia, is difficult to diagnose and has a high mortality. Here we determined the clinical characteristics and risk factors of histoplasmosis in people living with HIV (PLWH) in Pereira, Colombia.

**Materials and methods:**

This was a retrospective cross-sectional study (2014–2019) involving two tertiary medical centers in Pereira, Colombia. People hospitalized with HIV were included. Histoplasma antigen detection was performed in urine samples. Probable histoplasmosis was defined according to European Organization for Research and Treatment of Cancer/Invasive Fungal Infections Cooperative Group/National Institute of Allergy and Infectious Diseases Mycoses Study Group criteria.

**Results:**

172 HIV-infected patients were analyzed. Histoplasmosis was confirmed in 29% (n = 50/172) of patients. The logistic regression analysis showed that the risk factors for histoplasmosis were pancytopenia (OR 4.1, 95% CI 1.6–10.3, P = 0.002), < 50 CD4 + cells/μL (OR 3.1, 95% CI 1.3–7.3, P = 0.006) and Aspartate transaminase (AST) levels > 46 IU/L (OR 3.2, 95% CI 1.3–8, P = 0.010).

**Conclusions:**

Histoplasmosis is highly prevalent in hospitalized patients with HIV in Pereira, Colombia. The clinical findings are nonspecific, but there are some clinical abnormalities that can lead to suspicion of the disease, early diagnosis and prompt treatment. Urine antigen detection is useful for diagnosis, but is not widely available. An algorithmic approach is proposed for low-resource clinical settings.

## Background

Endemic mycoses are frequently found in Latin America where they represent a silent threat to public health. In Colombia, histoplasmosis is not a mandatory reporting entity, so, the burden of the disease is unknown. Despite its importance, clinical and epidemiological data remain scarce [1]. *Histoplasma capsulatum* is found primarily in soil containing bird excreta or bat guano. The disease begins when microconidia or small hyphal elements are inhaled and convert to yeasts in the lungs, or when organisms in previous quiescent foci of infection are reactivated during immunosuppression [1, 2]. *H. capsulatum* may cause a disseminated and potentially fatal disease in immunosuppressed patients (*Progressive Disseminated Histoplasmosis*) [1, 3].

The prevalence of histoplasmosis across the geography of Latin America is consistently high [2]. The disease has a wide spectrum of clinical manifestations and can be easily confused with miliary/extra-pulmonary tuberculosis [2]. The spectrum of clinical manifestations include fever (76.1%), cough (54.8%), constitutional symptoms (56.8%) and X rays abnormalities such as infiltrates (65.9%) and nodules (17.1%) [4]. There are other clinical findings such as anemia, lymphadenopathy, skin and mucosal lesions, hepatomegaly and splenomegaly. Therefore, a reliable diagnosis cannot be reached on the clinical findings alone [2, 4]. The detection of Histoplasma antigens in urine samples and real-time polymerase chain reaction (PCR) allows for an early diagnosis, reducing hospitalization costs and probably increasing survival [4, 5]. Detection of Histoplasma antigen in urine specimen by enzyme immunoassays (EIAs) has good diagnostic performance and has been extensively validated [6–9]. Different EIAs have been used as surrogate of histoplasmosis [6]. Histoplasma antigen capture ELISA developed at the Centers for Disease Control and Prevention (CDC) was validated in a cohort of AIDS patients in Guatemala [10] and also in Colombia, showing that it has a good diagnostic performance (86% sensitivity and 94% specificity) [11]. The enzyme-linked immunosorbent assay [Immuno-Mycologics (IMMY), Norman, OK, USA] was validated in two cohorts of people living with HIV (PLWH) from Guatemala and Colombia, the sensitivity was 98% and the specificity was 97% (cut off > 0.5 ng/ml) [6, 8].

A retrospective cross-sectional study was carried out aimed at establishing the clinical/laboratory characteristics, the prevalence and the risk factors for histoplasmosis in PLWH who were hospitalized in two tertiary centers in the city of Pereira, Colombia. The city is located in the Andean natural region of Colombia (the most populated natural region of Colombia). The patients in this study meet the criteria for *Probable Histoplasmosis* according to the *Consensus Definitions of Invasive Fungal Disease from the European Organization for Research and Treatment of Cancer and the Mycoses Study Group* (EORTC/MSG) [12, 13].

## Methods

### Study design and data collection

This was a retrospective cross-sectional study conducted in two tertiary centers in the city of Pereira, Colombia: the “San Jorge” University Hospital (SJUH) and the “Los Rosales” Clinic (LRC). The SJUH is a major public hospital in Pereira. It is equipped with over 200-beds. The LRC is a tertiary clinic equipped with over 100-beds. The study was approved by the Ethics Committee of the *Universidad Tecnológica de Pereira*. Patients hospitalized with an HIV diagnosis between May 2014 and April 2019 were identified from the database of both centers, followed by review of electronic patient medical records. All adult (≥ 18 years old) patients with documented HIV infection were candidates for inclusion in the study, if they were hospitalized for a clinical suspicion of an AIDS-defining disease like histoplasmosis based on clinical manifestations and laboratory findings such as the following: fever and/or weight loss (> 10% of usual body weight), cough, diarrhea, miliary opacities on thorax imaging, pancytopenia, lymphadenopathy, splenomegaly and/or hepatomegaly and abnormal liver function test, ferritin and lactic dehydrogenase (LDH) serum levels. The presence of co-infections and AIDS-defining conditions was established. Demographic and clinic information were extracted using standardized data collection sheets.

#### Case definition

A case of histoplasmosis was defined as an HIV-patient who had a urinary antigen elevated above the cut-off point of the reference laboratory and/or histological findings of mycotic forms compatible with *H. capsulatum* in tissue biopsies*.* The diagnosis of histoplasmosis was made based on the recommendations of the EORTC/MSG [12, 13]. Patients with incomplete medical records and pregnant women were excluded. PLWH with clinical suspicion of histoplasmosis that tested negative for Histoplasma urinary antigen and did not have histological findings of mycotic forms were classified as *no histoplasmosis group*.

#### Laboratory methods

Specimens analyzed included blood, urine, lymphatic nodes or gastrointestinal biopsies depending on the patient’s presentation. All tissue biopsies were stained with Giemsa, Gram, and Ziehl–Nielsen and were cultured for bacteria, mycobacteria, and fungi. The culture medium for fungi used were Mycosel and Sabouraud. Histoplasma antigen detection in urine was performed, mostly, by IMMY's antigen enzyme immunoassay, in a reference laboratory. Mycobacterium tuberculosis was detected with Ziehl–Neelsen stain, culture or PCR. In some patients, the presence of *Cryptococcus* spp was evaluated, when appropriate, by India ink stain and Cryptococcus latex agglutination test. Other laboratory tests included complete blood cell count, liver function tests, renal function tests, ferritin levels, CD4 + cell count, HIV viral load, and LDH levels. Chest X-rays and chest CT scans were performed, as well as abdominal ultrasound and abdominal CT scan, when appropriate.

#### Statistical analysis

We used descriptive statistics to analyze and report data. The Kolmogorov–Smirnov test was performed to assess for normal distribution. Baseline characteristics were compared using Chi-square or Fisher’s exact tests, when appropriate, for categorical variables. For continuous data, the assumptions of normality were verified and for those that fulfilled them, Student’s *T* tests were performed. Non-parametric tests were used for those that did not fulfill the assumptions of normality (Wilcoxon or Man Whitney’s *U*). A logistic regression analysis was constructed. All associations were considered significant for a value of P < 0.05. Variables with P < 0.10 were included in the model and remained in the final model if P < 0.05. IBM SPSS Statistics software version 20 was used for all statistical analyses. The diagnostic performance of risk factors for histoplasmosis in PLWH was determined in terms of sensitivity, specificity, positive predictive value (PPV), negative predictive value (NPV), positive likelihood ratio (+ LR) and negative likelihood ratio (− LR).

## Results

### Clinical and laboratory characteristics.

**Fig. 1:**
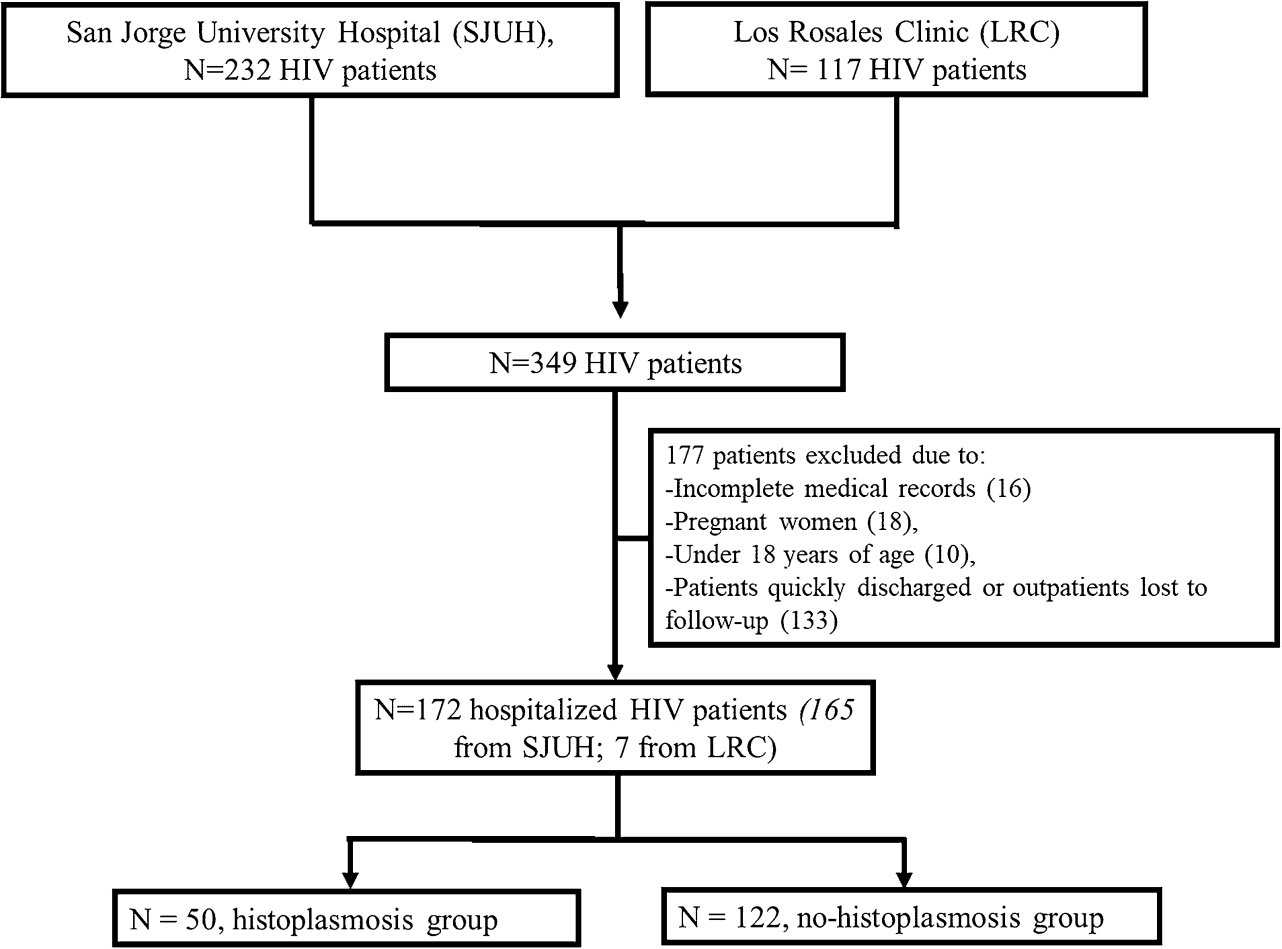
Flow chart for patient selection from study centers

Histoplasmosis was suspected in 172 patients based on their clinical and laboratory characteristics. All inpatients were evaluated by an internal medicine physician or an infectious diseases physician. All patients underwent EIAs to detect *H. capsulatum* polysacharide antigen in urine and 29% (n = 50) of them were diagnosed as histoplasmosis based on the EORTC/MSG statements [12, 13]. One patient had findings of fungal forms of *H. capsulatum* in peripheral blood smear. Another three patients had a report of fungal forms of *H. capsulatum* in colon biopsies. A total of 122 (70%) patients were negative for Histoplasma antigen in urine (no histoplasmosis group). PCR tests for histoplasmosis were not performed as they were not available in our area. Figure 1 shows the flow chart for patient selection from study centers.

The mean age was 41 years old, 76% were men, and 79% came from urban areas. Half of the patients had HIV de novo and only 16% of the patients had received antiretroviral treatment in the past. Most of the patients were in AIDS category 3 (87%) and C (77%). The CD4 + cell count was lower in the histoplasmosis group compared to the “no histoplasmosis” group (28 cells/μL, IQR 14–52 vs 73 cells/μL, IQR 30–137, P = 0.002). AIDS Category 3 was more frequent in the histoplasmosis group than in the no histoplasmosis group (95% vs 84%, P = 0.038). Other AIDS-defining conditions were prevalent, mainly Tuberculosis and Toxoplasmosis. There was only one case of Cryptococcosis. Toxoplasmosis was more frequently found in the no histoplasmosis group (10% vs 23%, P value = 0.039). The histoplasmosis *group* was more likely to receive antifungal therapy (94% vs 65.6%, P = 0.00) primarily based on amphotericin B deoxycholate, without significant increase of episodes of acute kidney injury (38% vs 31%, P value = 0.44). There was no difference in terms of in-hospital death (28% vs 23%, P = 0.56). The H-Score was higher in the histoplasmosis *group* (71 points, IQR 40–95, vs 42 points, IQR 37–75, P = 0.041), but there were no confirmed cases of hemophagocytic lymphohistiocytosis. There were no cases of viral hepatitis.

Regarding the clinical manifestations, there were no differences between groups. Only headache was more frequently found in the no histoplasmosis group (22% vs 44%, P = 0.006), probably explained by the higher prevalence of toxoplasmosis in the no histoplasmosis group. Some laboratory abnormalities were more common in the histoplasmosis *group* compared to the *no histoplasmosis group *(see Table 1 for clinical and laboratory characteristics).

**Table 1:** Demographic, clinical and laboratory characteristics

	Histoplasmosis group, n = 50 (%)	No histoplasmosis group, n = 122 (%)	P value
Male %	39(78)	92(75.4)	0.7
Age in years, median (IQR)	35 (29–46)	40 (29–54)	0.2
Days of hospital stay, median (IQR)	32 (17–48)	23 (14–38)	0.25
In-hospital mortality	14 (28)	29 (23)	0.56
Viral load (copies/mL), median (IQR)	289,000 (76,300–1,310,951)	265,454 (63,763–773,753)	0.12
CD4 (cells/μL), median (IQR)	28 (14–52)	73 (30–137)	0.002
< 50 CD4 cells/μL, %	36 (73.4)	47 (40.8)	0.001
AIDS category 1	0	5 (4.09)	0.32
AIDS category 2	2 (4.08)	13 (11)	0.23
AIDS category 3	47 (95)	97 (84)	0.03
AIDS category C	50 (100)	84 (68)	0.001
Antifungal treatment	47(94)	80 (65)	0.001
Other AIDS-related co-infections			
Tuberculosis	22 (44)	57 (46)	0.7
Pneumocystis pneumonia	7 (14)	17(13)	0.9
Toxoplasmosis	5 (10)	29 (23)	0.03
Symptoms and clinical characteristics			
Constitutional symptoms (weight loss, night sweats, with/without fever)	44 (88)	105 (86)	0.73
Unintended weight loss	43 (86)	97 (79)	0.32
Fever	40 (80)	89 (73)	0.33
Diarrhea	33 (66)	75 (61)	0.58
Cough	28 (56)	78 (63)	0.33
Dyspnea	21 (42)	67 (54)	0.12
Lymphadenopathy	22 (44)	63 (51)	0.36
Headache	11 (22)	54 (44)	0.006
Mucocutaneous lesions	16 (32)	49 (40)	0.31
Shock and multiple organ dysfunction syndrome	13 (26)	18 (14)	0.08
H-Score, median (IQR)	71 (40–95)	42 (37–75)	0.04
Laboratory abnormalities and imaging tests			
Ferritin levels > 1000 ng/mL	28 (77)	39 (56)	0.03
ALP levels (IU/L). median (IQR)	366 (226–802)	173 (100–326)	0.003
AST levels > 46 IU/L	36 (80)	52 (49)	0.001
AST/ALT ratio > 2	20 (40)	22 (20)	0.003
Leukocyte count in CBC (10^9^/L), median (IQR)	3.3 (1.9–5.8)	5.7 (3.8–7.8)	0.001
Platelet count in CBC (10^9^/L), median (IQR)	131 (56–230)	230 (156–323)	0.009
Pancytopenia	21 (42)	14 (11)	0.001
Abnormal chest CT	38 (88)	66 (68)	0.014

*IQR* interquartile range, *ALP* alkaline phosphatase, *AST* aspartate transaminase, *ALT* alanine transaminase, *CBC* complete blood count, *CT* computed tomography

### Logistic regression to establish risk factors for histoplasmosis

The multivariable logistic regression analysis included the variables with P-value of < 0.10 (see Table 1). The risk factors for histoplasmosis were pancytopenia (OR 4.1, 95 CI 1.6–10.3, P = 0.002), < 50 CD4 + cells/μL (OR 3.1, 95 CI 1.3–7.3, P = 0.006) and Aspartate transaminase (AST) levels > 46 IU/L (OR 3.2, 95 CI 1.3–8, P = 0.01). Pancytopenia showed the highest specificity and a high NPV (88% and 78% respectively), a low sensitivity (42%) and the highest PPV (60%). No other tests showed good specificity. This test showed a + LR of 3.65. The AST levels > 46 IU/L showed a high sensitivity. See Tables 2 and 3.

**Table 2:** Risk factors for the diagnosis of histoplasmosis

Variables	OR	P	95 CI
Pancytopenia	4.135	0.002	1.648–10.376
< 50 CD4 cells/μL	3.195	0.006	1.398–7.303
AST levels > 46 IU/L	3.265	0.010	1.332–8.004

In the logistic regression analysis, Omnibus tests of model coefficients had a P value of 0.001; Nagelkerke’s R was 29% (0.290); the Hosmer and Lemeshow test showed a P value of P = 0.89

**Table 3:** Diagnostic performance of clinical variables for the diagnosis of histoplasmosis

	Pancytopenia	< 50 CD4 cells/μL	AST levels > 46 IU/L	< 200 CD4 cells/μL	Elevated AST plus < 200 CD4 cells/μL	Pancytopenia plus < 50 CD4 cells/μL
Sensitivity	42	73.5	80	95.9	100	84
Specificity	88.5	59.1	50.5	15.7	11.7	57.4
Positive predictive value	60	43.4	40.9	32.6	32.1	44.7
Negative predictive value	78.8	84	85.5	90	100	89.7
Positive likelihood ratio	3.65	1.8	1.62	1.14	1.13	1.97
Negative likelihood ratio	0.66	0.45	0.4	0.26	0	0.28

A less than 200 CD4 + cells/μL (AIDS category 3) was more frequently found in histoplasmosis group than no histoplasmosis group (95% vs 84%, P = 0.038). In an exploratory way, having < *200 CD4* + cells/μL showed the highest sensitivity and a high NPV (> 90%) for the diagnosis of histoplasmosis. For these reasons we decided to explore the performance of two combined variables (in order to find simple strategies to reach a presumptive diagnosis of histoplasmosis in low-resource contexts). *Elevated AST plus* < *200 CD4* + cells/μL showed a sensitivity and NPV of 100%, with 0% false negatives, but the specificity was very low (11%) and the false positives very high (88%), implying that the combination of both tests are only useful to rule out histoplasmosis if the patient has > 200 CD4 + cells/μL and normal AST transaminase levels. The combination of *Pancytopenia plus* < *50 CD4* + cells/μL showed a sensitivity of 84%, but a low specificity (57%). None of the tests analyzed has a good balance between sensitivity and specificity. The only variable with a good + LR and good specificity was pancytopenia. See Tables 2 and 3.

## Discussion

We had described the main characteristics of histoplasmosis in hospitalized PLWH. The incidence of histoplasmosis was very high, the patients included in the study had very low rates of self-awareness of their HIV status and prior anti-retroviral treatment. The viral load was very high in both groups and they were quite immunosuppressed, reflecting a very long course of HIV disease. It became clear that there is an inversely proportional relationship between the CD4 + cell count and the incidence of histoplasmosis [14, 15]. This is seen, mainly, with < 200 CD4 + cells/μL, and it's even worse if the CD4 + cell count falls below 50 cells/μL.

The spectrum of symptoms and laboratory abnormalities that we found are similar to that reported elsewhere [4, 16, 17]. *Caceres *et al. showed that PLWH with histoplasmosis have increases in transaminases and a decrease in hemoglobin concentration [18]. *Samayoa *et al. found that CD4 + cell count, platelet count and hemoglobin levels are significantly lower in histoplasmosis-cases than non-cases. On the other hand, LDH levels, serum AST levels, bilirubin levels and alkaline phosphatase (ALP) were significantly higher in histoplasmosis-cases than non-cases [16]. *Falci *et al. in Brazil demonstrated that < 50 CD4 + cells/μL, pancytopenia, miliary pattern on thorax imaging, hepatomegaly, generalized lymphadenopathy and LDH > 1000 IU/L, are factors that may aim for the prediction of Probable/Proven histoplasmosis [19].

**Fig. 2:**
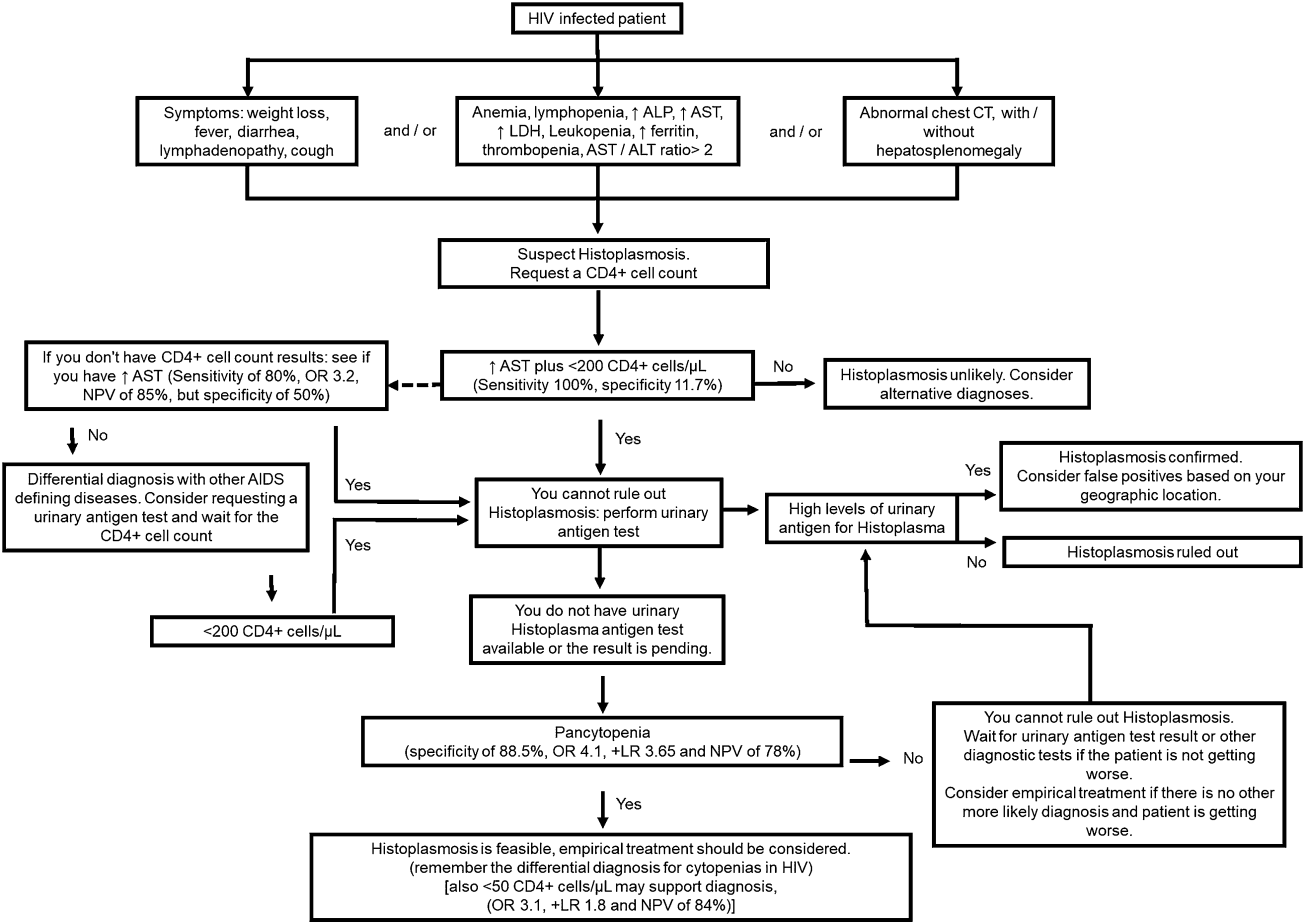
Algorithmic approach for the diagnosis and treatment of PLWH with suspected AIDS-defining conditions such as histoplasmosis, for low-resource clinical scenarios. *ALP* alkaline phosphatase, *AST* aspartate transaminase, *ALT* alanine transaminase, *CT* computed tomography, +* LR* positive likelihood ratio, *NPV* negative predictive value

Histoplasmosis must be recognized and treated promptly, unfortunately the main symptoms, signs and laboratory abnormalities that could raise its suspicion are nonspecific. The clinical and laboratory characteristics found by us are somewhat consistent with what has been reported elsewhere [16, 18, 19], therefore, this prompts us to believe that it is feasible to make an algorithmic approach that allows a timely presumptive diagnosis of histoplasmosis in hospitalized PLWH with suspicion of an AIDS defining condition (in endemic regions for histoplasmosis), when there are limitations, mainly economic, to perform timely diagnostic tests. Figure 2 shows a proposed algorithmic approach for the early diagnosis and treatment of histoplasmosis. This could be useful in low- and middle-income countries, where access to laboratory tests is limited, as in Colombia, where the detection of urinary antigen by EIAs for histoplasmosis is not funded by the population’s health insurance system.

### Conclusions

Histoplasmosis is a common opportunistic infection in hospitalized PLWH and is associated with high mortality [1, 2, 20, 21]. The risk factors for Probable/Histoplasmosis were pancytopenia, < 50 CD4 + cells/μL and high AST levels. The hidden burden of the histoplasmosis will continue until we reach a successful implementation of early diagnosis and treatment strategies for HIV. An algorithmic diagnostic approach could be useful to make a timely presumptive diagnosis and provide early empiric treatment in endemic areas when economic resources and diagnostic tests are scarce.

This study had some limitations. First, the results were obtained retrospectively from two centers in the same city, which may limit the generalization of the results to a more extensive geographic context, however our data are in line with other reported analyses. Second, we concentrate our analysis to a mainly AIDS population, therefore the extrapolation of the results to immunosuppressive diseases other than HIV may not be suitable. Third, the EORTC/MSG expert consensus [12, 13] warns that urinary Histoplasma antigen supports a diagnosis of probable endemic mycosis, in conjunction with appropriate host and clinical criteria, but cannot be considered sufficient evidence of proven histoplasmosis because there are cross reactions of the urinary antigen test with infections caused by *Paracoccidioides brasiliensis, Blastomyces dermatitidis, Coccidioides immitis*, and *Penicillium marneffei* [22]. However, Blastomycocosis, Coccidioidomycosis and the disease caused by *Penicillium marneffei* are not endemic in Colombia and belong to other geographical areas [1, 23, 24]. Paracoccidioidomycosis is endemic in Colombia but has a very low prevalence in PLWH [1].

## Data Availability

The datasets used and/or analyzed during the current study are available from the corresponding author on reasonable request.

## Abbreviations

SJUH: San Jorge University Hospital
EIAs: Enzyme immunoassays
PLWH: People living with HIV
RT-PCR: Real-time polymerase chain reaction
CLSI: Clinical & laboratory standards institute
ALP: Alkaline phosphatase
ALT: Alanine transaminase
AST: Aspartate transaminase
CBC: Complete blood count
LDH: Lactate dehydrogenase
+ LR: Positive likelihood ratio
− LR: Negative likelihood ratio
